# Clinical relevance of total choline (tCho) quantification in suspicious lesions on multiparametric breast MRI

**DOI:** 10.1007/s00330-020-06678-z

**Published:** 2020-02-17

**Authors:** Claudia Sodano, Paola Clauser, Matthias Dietzel, Panagiotis Kapetas, Katja Pinker, Thomas H. Helbich, Alexander Gussew, Pascal Andreas Baltzer

**Affiliations:** 1 Department of Biomedical Imaging and Image-guided Therapy, Division of Molecular and Gender, Imaging, Medical University of Vienna, Waehringer-Guertel 18-20, A-1090 Vienna, Austria; 2 Institute of Radiology, Universitätsklinikum Erlangen, Maximiliansplatz 1, 91054 Erlangen, Germany; 3 Department of Radiology, Breast Imaging Service, Memorial Sloan Kettering Cancer Center, 300 E 66th Street, New York, NY 10065 USA; 4 Universitätsklinik und Poliklinik für Radiologie, Ernst-Grube-Str. 40, D-06120 Halle (Saale), Germany

**Keywords:** Magnetic resonance spectroscopy, Magnetic resonance imaging, Breast neoplasms, Prognosis, Sensitivity and specificity

## Abstract

**Purpose:**

To assess the additional value of quantitative tCho evaluation to diagnose malignancy and lymph node metastases in suspicious lesions on multiparametric breast MRI (mpMRI, BI-RADS 4, and BI-RADS 5).

**Methods:**

One hundred twenty-one patients that demonstrated suspicious multiparametric breast MRI lesions using DCE, T2w, and diffusion-weighted (DW) images were prospectively enrolled in this IRB-approved study. All underwent single-voxel proton MR spectroscopy (^1^H-MRS, point-resolved spectroscopy sequence, TR 2000 ms, TE 272 ms) with and without water suppression. The total choline (tCho) amplitude was measured and normalized to millimoles/liter according to established methodology by two independent readers (R1, R2). ROC-analysis was employed to predict malignancy and lymph node status by tCho results.

**Results:**

One hundred three patients with 74 malignant and 29 benign lesions had full ^1^H-MRS data. The area under the ROC curve (AUC) for prediction of malignancy was 0.816 (R1) and 0.809 (R2). A cutoff of 0.8 mmol/l tCho could diagnose malignancy with a sensitivity of > 95%. For prediction of lymph node metastases, tCho measurements achieved an AUC of 0.760 (R1) and 0.788 (R2). At tCho levels < 2.4 mmol/l, no metastatic lymph nodes were found.

**Conclusion:**

Quantitative tCho evaluation from ^1^H-MRS allowed diagnose malignancy and lymph node status in breast lesions suspicious on multiparametric breast MRI. tCho therefore demonstrated the potential to downgrade suspicious mpMRI lesions and stratify the risk of lymph node metastases for improved patient management.

**Key Points:**

*• Quantitative tCho evaluation can distinguish benign from malignant breast lesions suspicious after multiparametric MRI assessment.*

*• Quantitative tCho levels are associated with lymph node status in breast cancer.*

*• Quantitative tCho levels are higher in hormonal receptor positive compared to hormonal receptor negative lesions.*

**Electronic supplementary material:**

The online version of this article (10.1007/s00330-020-06678-z) contains supplementary material, which is available to authorized users.

## Introduction

Breast cancer is a major burden on the female population and subsequently for the socioeconomic system. Consequently, most industrialized nations have introduced nation-wide mammography screening programs that are supported by the majority of specialist societies [1]. Screening with mammography and additional evaluations with ultrasound and MRI add in cancer diagnosis and assessment of disease [2–4].

Currently, breast cancer is treated by a combination of surgery, pharmaceutical therapy, and radiation therapy tailored to the individual patient based on risk factors including cancer type, lymph node metastases, and menopausal status [5]. To select the best treatment option, breast cancer needs to be diagnosed and accurately characterized. Although breast cancer type can be determined using image-guided biopsies, biopsies only provide information on the biopsied part of the lesion which is subject to selection bias, potentially leading to inaccurate diagnoses. It has been demonstrated that imaging characteristics, in particular those derived from breast MRI, can help to identify prognostically relevant information [6–11]. Lymph node status is more challenging to assess: metastatic lymph nodes might be present even if suspicious imaging characteristics are absent [12–14], and in some cases of lymph nodes with uncertain morphology it might be difficult to perform a biopsy and confirm or exclude metastasis. Thus, there is a need for more accurate and non-invasive methods to determine lymph nodes status before treatment.

Proton MR spectroscopy (^1^H-MRS) allows the assessment of total choline (tCho), a compound resonance that is connected to multiple enzymatic changes involved in oncogenesis, tumor progression, and metastasis [15]. ^1^H-MRS has therefore been proposed as an additional tool to improve lesion characterization, but its use has been restricted due to the technical difficulties related to data acquisition and interpretation, yielding heterogeneous results [16]. Nevertheless, ^1^H-MRS is able to give information on a molecular level without the use of contrast agents, and its feasibility is highly improved owing to the improved performance of magnets and coils. Accurate breast cancer diagnosis including lymph node status is pivotal in determining treatment and currently requires biopsies and open surgery. We hypothesize that the additional molecular information provided by ^1^H-MRS can be used to facilitate breast lesion workup by potentially avoiding invasive diagnostic procedures to diagnose breast cancer and lymph node metastases.

The aim of this study was to evaluate the additional value of quantitative tCho evaluation to diagnose malignancy, breast cancer type, and lymph node status in suspicious lesions on multiparametric breast MRI (mpMRI, BI-RADS 4, and BI-RADS 5).

## Methods

### Study design, participants, and reference standard

This prospective, IRB-approved single-center cross-sectional study was performed at the university hospital of Jena, Germany, a certified tertiary care academic breast center. The study aimed to evaluate the additional value of tCho measurements from ^1^H-MRS to diagnose malignancy, breast cancer type, and lymph node status in MRI BI-RADS 4 and BI-RADS 5 lesions. A summary of the study design is given in Supplementary figure 1. Eligible were consecutive patients undergoing breast MRI for further workup of equivocal and suspicious conventional imaging (digital mammography, MG; ultrasound, US) findings (BI-RADS 0, BI-RADS 4, and BI-RADS 5). To be further eligible for this study, they had to present with a contrast-enhancing lesion of at least 8 mm in size on the MRI scan that was rated as suspicious (MRI BI-RADS 4 and BI-RADS 5) according to pre-defined criteria: non-circumscribed or spiculated margins, type II or III curve, non-circumscribed, non-hyperintense T2w correlate, and intermediate to low apparent diffusion coefficient (ADC) values < 1.5 10^−3^ mm^2^/s were considered suspicious, the combined appearance of several of these criteria as highly suspicious. This initial assessment was done by one of two breast MRI experts (W.A.K., P.A.B.) while the patient was placed in the scanner and MR spectroscopy was performed in eligible patients in the same breast MRI examination. Exclusion criterion was missing informed consent. Note that ineligibility for contrast-enhanced MRI was not counted as an exclusion criterion as patients were recruited before the MRI examination at the day of their visit while claustrophobia, presence of allergies, and metallic implants were excluded when scheduling the MRI appointment. Patients were excluded from the final analysis in case of termination of the examination before completion, incomplete imaging data, or non-interpretable imaging data due to artifacts (e.g., caused by motion).

Histopathologic workup of the patients served as the reference standard and was always performed after the breast MRI scan. Spectroscopy results were not used to guide patient management decisions. Biopsies were performed either US-guided by 14-gauge core needle biopsy in case of US visibility or MRI-guided 9G console-mounted vacuum-assisted biopsy in case the lesion was not visible on conventional imaging. All malignant lesions underwent surgery. If there was a discordance between biopsy and surgery, the latter was used as reference standard. However, if the patient was treated by neoadjuvant therapy, the histopathological result from the biopsy was used as reference standard. Benign results were checked for consistency within weekly interdisciplinary meetings and re-biopsied or operated on when deemed necessary (inconclusive results, e.g., B1 or unspecific B2 in case of circumscribed lesions). Lesions with uncertain malignant potential (B3) upon histopathology were surgically removed [17]. Benign B2 findings deemed consistent with histopathology were followed up by the imaging test best suited to visualize the lesion (either MG, US, or MRI) over 24 months as has been done in prior studies (e.g., listed in [18]). At the time of the study, sentinel lymph node biopsy (SNLB) was performed in all surgeries of malignant lesions. In case of positive SNLB, axillary lymph node dissection was performed. For the lymph node analysis in this study, only patients that underwent axillary surgery without prior neoadjuvant therapy were used. The study design is summarized in a flowchart (Supplementary Fig. 1).

### MR imaging and proton MR spectroscopy

All imaging was performed on a 1.5-T scanner (Siemens Magnetom Sonata, Siemens Healthineers) using the dedicated vendor-supplied 4-channel double breast coil. The standardized protocol was in accordance with international recommendations and employed an axial 2D T2-w turbo spin echo (TSE, TR 8900 ms, TE_eff_ 207 ms, field of view 340 mm, matrix 512 mm, slice thickness 3 mm) and a dynamic T1-weighted spoiled gradient echo sequence (fast low angle shot, FLASH, GRAPPA factor 2, TR 113 ms, TE 5 ms, FOV 340 mm, matrix 384, slice thickness 3 mm). Afterwards, 0.1 mmol/l body weight of gadopentetate dimeglumine (Gd-DTPA, Magnevist) was administered intravenously as a rapid bolus (3 ml/s), performed by an automatic injector (Spectris, Medrad), followed by 20 ml saline solution. The dynamic scan had a temporal resolution of 1 min and was repeated 8 times. An injection delay of 30 s between scans 1 (precontrast) and 2 (first postcontrast acquisition) was applied. In addition, a diffusion-weighted Echo Planar Imaging (EPI) sequence (GRAPPA factor 2, TR 3500, TEeff 80, echo distance 0.95 ms, six averages, three b-values: 0, 750, 1000 s/mm^2^, spectral fat saturation, in plane resolution 1.8 × 1.8 mm, slice thickness 6 mm) was acquired. Apparent diffusion coefficient (ADC) maps were calculated in-line by the scanner software.

Proton MR spectroscopy (^1^H-MRS, vendor-supplied standard single-voxel point-resolved spectroscopy sequence PRESS, TR 2000 ms, TE 272 ms, vector size 1024, weak water suppression with a bandwidth of 35 Hz, 128 averaged acquisitions with an acquisition time of 4:16 min) was acquired after automatic and manual first- and second-order shim gradient adjustments. The full width at half maximum was usually below 25 Hz. An additional scan without water suppression using 32 averages and the same adjustments was subsequently acquired as an internal reference. Including planning and shimming, spectroscopy was performed within 8–10 min. All spectra acquisitions were performed by an experienced breast radiologist trained in MR spectroscopy and acquisition (referred to as investigator, > 10 years of experience, P.A.B.) and supervised by two dedicated spectroscopists (> 25 and > 10 years of experience, A.G., R.Z.).

### Data analysis

All acquired spectra were technically reviewed by the investigator after acquisition using the vendor-supplied software and then exported as raw data (.rda file) for further analysis. Consecutive analysis by two independent readers (both radiologists with > 2 years of training, supervised by the investigator) was done using v 4.0 of the free java Magnetic Resonance User Interface (jMRUI) software (www.jmrui.eu). The readers knew the study design but were not aware of any specific patient-related data. FID data were zero filled to 2048 data points and a Gaussian apodization of 5 Hz was applied. Fourier transformation and manual phase correction followed. The spectra were referenced using the water and methylene peaks as reference (4.74 and 1.33 ppm). Quantification was done by the AMARES algorithm [19] using prior knowledge on peak position (soft constraints ± 0.15 ppm) and tCho peak amplitude (soft constraints 0–200 arbitrary units). The fitting result was compared to the original spectrum by subtraction and was considered acceptable if systematic baseline deviations > 1.5-fold higher the baseline noise were absent. In case of an insufficient quantitation of tCho at 3.23 ppm, soft constraints for peak amplitude were adapted based on overall noise level and visual inspection of visible tCho peaks. The fitting procedure was repeated until the subtracted peak yielded a zero baseline as defined above. Absolute tCho concentrations were quantified in mmol/l by using the following equation:

$$ {C}_{\mathrm{Cho}}=\frac{I_{\mathrm{cho}}}{I_{\mathrm{wat}}}\cdotp \frac{N_{\mathrm{wat}}^H}{N_{\mathrm{cho}}^H}\cdotp \frac{R_{\mathrm{wat}}}{R_{\mathrm{Cho}}}\cdotp {C}_{\mathrm{wat}} $$

$$ {R}_x={\mathrm{e}}^{-\frac{\mathrm{TE}}{T_2^x}}\cdotp \left(1-{\mathrm{e}}^{-\frac{\mathrm{TR}}{T_1^x}}\right) $$

While the water concentration was approximated to 55,600 mmol/l, the hydrogen quantities in water and choline molecules were set to 2 and 9, respectively. Finally, *T*_1_ and *T*_2_ relaxation times of water and choline were adapted from a review article of Haddadin et al (${T}_1^{\mathrm{wat}}$ = 0.441 s; ${T}_1^{\mathrm{Cho}}$ = 1.513 s; ${T}_2^{\mathrm{wat}}$ = 0.075 s; ${T}_2^{\mathrm{Cho}}$ = 0.269 s) [20, 21].

In addition, lesion size was measured volumetrically by assessing lesion dimensions in all three plains on early DCE images acquired 2 min after CM injection. The lesion volume was calculated in milliliters by multiplying the lesion size in all three plains in centimeters with 0.52 and expressed in milliliters.

### Statistical analysis

Continuous variables are presented by median, interquartile range, and range. Quantitative tCho values were calculated according to the formula above and compared between lesion subgroups using non-parametric statistics (Mann-Whitney *U* test in case of two independent samples, Kruskal-Wallis test in case of > 2 independent samples). Bland-Altman statistics were performed to calculate mean differences and limits of agreement between both tCho measurements of R1 and R2.

The area under the receiver operating characteristics (ROC) curve (AUC) was used as a measure of diagnostic performance for tCho measurements. Two independent sets of ROC analyses were performed for R1 and R2, respectively: diagnosis of benign vs malignant and diagnosis of lymph node status (negative vs positive) in malignant lesions. The null hypothesis was defined as AUC = 0.5. To determine exploratory tCho cutoff values, we followed the approach of identifying an exploratory “rule-out” criterion for malignancy [22, 23]. Such a criterion was considered present if the corresponding tCho cutoff yielded a sensitivity of ≥ 95% in both readers. As all lesions in this study were considered suspicious by mpMRI (T2w; diffusion-weighted imaging, DWI; dynamic contrast enhanced, DCE), the specificity value at this cutoff indicates the rate of benign lesions that may not have required biopsy if the tCho value was below this exploratory threshold. *P* values of < 0.05 were considered statistically significant.

All statistical analyses were performed using Medcalc 18.10.2 software.

## Results

### General

One hundred twenty-one patients were examined with the study protocol. Eighteen of these (14.9%) had to be excluded due to technically insufficient spectra (bulk motion during acquisition and wrongly applied water suppression in the reference scan being the most common reasons). Finally, 103 patients had full diagnostic and reference data and were eligible for further analysis (median age 55 years, range 23–83 years) with 103 lesions (74 malignant, 29 benign, lymph node status was positive in 17 and negative in 46 breast cancers; in 11 patients no reliable reference standard for axillary lymph node status was available due to neoadjuvant therapy before surgery. See Table 1 for histological details and Supplementary figure 1 for the patient selection flowchart). Lesion volumes ranged from 0.45 to 274.6 ml with a median volume of 3.98 ml. No significant differences were found between median volumes of benign (3.59 ml, range 0.47–61.92 ml) and malignant (4.06, range 0.45–274.6 ml) lesions (*p* = 0.401, Mann-Whitney *U* test). Spectroscopy voxel sizes ranged between 1.73 and 27 ml with a median volume of 5.83 ml.

**Table 1:** Lesion characteristics

Lesions characteristics			tCho (R1, mmol/kg)^#^	tCho (R2, mmol/kg)^#^	*p* value
Malignant lesion histological subtypes		*n* = 74	3.22 (2.07, 7.17; 0.23–15.89)	3.29 (1.83, 7.28; 0.22–16.00)	
	Invasive ductal carcinoma NST	57	2.82 (2.01, 7.32; 0.23–15.89)	2.95 (1.78, 7.32; 0.22–16.00)	0.346 (R1), 0.301 (R2)
	Invasive lobular carcinoma	12	4.36 (2.66, 7.51; 1.04–11.19)	4.20 (2.94, 8.45; 1.41–11.01)	
	Ductal carcinoma in situ	2	3.57 (3.03, 4.12; 3.03–4.12)	3.29 (3.26, 3.32; 3.26–3.32)	
	Other^§^	3	1.79 (0.99, 2.87; 0.73–3.23)	1.96 (1.03, 2.68; 0.72–2.93)	
Malignant lesion grading		*n* = 74			
	G1	5	1.65 (1.07, 4.79; 0.62–7.79)	1.74 (1.01, 5.50; 0.49–9.05)	0.200 (R1), 0.264 (R2)
	G2	26	3.57 (2.65, 7.41; 0.23–15.89)	3.70 (2.67, 7.62; 0.22–16.00)	
	G3	43	3.11 (1.82, 6.30; 0.54–13.22)	3.10 (1.75, 6.33; 0.83–13.17)	
Malignant lesion receptor status		*n* = 73*			
	HR+, her2neu−	38	4.42 (2.62, 7.94; 0.23–15.89)	4.25 (2.66, 4.27; 0.22–16.0)	0.051 (R1), 0.056 (R2)^x^
	HR+, her2neu+	15	3.20 (2.67, 4.09; 1.70–7.32)	2.95 (2.66, 4.27; 1.41–8.73)	
	HR−, her2neu+	6	2.41 (1.58, 2.48; 1.33–4.00)	2.37 (1.51, 2.42; 1.29–3.71)	
	HR−, her2neu−	14	1.95 (1.69, 6.47; 0.84–11.19)	1.81 (1.70, 6.74; 0.83–11.01)	
Lymph node status		*n* = 63*			
	LN+	17	7.17 (3.78, 8.43; 2.48–13.22)	8.19 (3.81, 8.81; 2.42–13.17)	0.002 (R1), < 0.001 (R2)
	LN−	46	2.79 (1.75, 2.66; 0.23–15.89)	2.86 (1.71, 4.92; 0.22–16.00)	
	Benign lesions	*n* = 29	0.93 (0.27, 2.55; 0.00–6.00)	1.13 (0.27, 2.57; 0.00–6.06)	
	Epithelial proliferations	15	1.07 (0.13, 2.94; 0.00–6.00)	1.10 (0.11, 2.89; 0.00–6.06)	0.919 (R1), 0.872 (R2)
	Fibroadenoma	8	1.13 (0.09, 3.37; 0.00–4.78)	1.13 (0.09, 3.59; 0.00–5.03)	
	Solid B3^$^	3	1.93 (0.30, 2.16; 0.30–2.16)	2.11 (0.41, 2.22; 0.41–2.22)	
	Inflammation	3	0.73 (0.26, 0.93; 0.26–0.93)	0.84 (0.25, 1.13; 0.25–1.13)	

* in 11 patients, no reliable reference standard for axillary lymph node status was available due to neoadjuvant treatment before surgery and in one patient no receptor status was available in a DCIS; ^#^given as: median (interquartile range Q25, Q75, range); ^§^one invasive papillary, mucinous, and tubular carcinoma, respectively; ^$^two papilloma, one phyllodes; ^x^HR+ breast cancers differed significantly from all others (*p* = 0.018 (R1) and *p* = 0.020 (R2), respectively

### tCho levels in benign and malignant lesions

tCho levels were significantly higher in malignant compared to benign lesions (*p* < 0.001 for both R1 and R2, respectively, see Table 1 and supplementary figure 2). While highest tCho levels were observed in ILC (see Table 1), these differences compared to other cancer subgroups did not prove statistically significant (*p* = 0.346, R1; *p* = 0.301, R2). Lower tCho was observed in G1 as compared to G2 and G3 cancers (Table 1), again without demonstrating statistical significance (*p* = 0.200, R1; *p* = 0.264, R2).

**Fig. 1:**
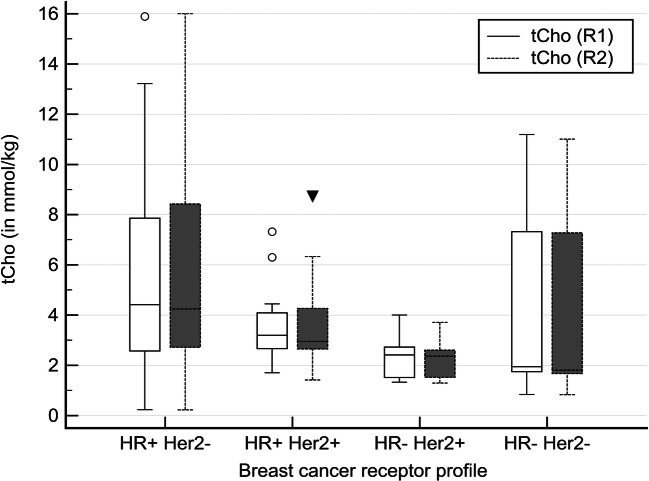
Boxplots of tCho results stratified by breast cancer receptor status (HR: hormonal receptors, Her2+: her2neu receptor status, + indicating positivity, − negativity). The observed tCho levels were significantly higher in hormonal receptor positive breast cancers (*p* = 0.018, R1; *p* = 0.020, R2)

Full receptor status was available in 73 of 74 malignant lesions (Table 1). Figure 1 shows tCho levels stratified by breast cancer receptor status. The tCho levels in hormonal receptor positive breast cancers were significantly higher than those in hormonal receptor negative lesions (*p* = 0.018, R1; *p* = 0.020, R2). No further subgroup differences between breast cancer subgroups as distinguished by receptor status were found (*p* = 0.051, R1 and *p* = 0.056, R2).

In 63 cancer patients with available lymph node status (11 without reference standard after neoadjuvant breast cancer treatment), tCho levels were significantly higher in those with positive as compared to negative lymph nodes (*p* = 0.002, R1; *p* < 0.001, R2, Table 1).

In benign lesions, no significant subgroup differences were found (*p* = 0.919, R1; *p* = 0.872, R2; see Table 1).

### Reproducibility of tCho quantification on acquired spectroscopy data

**Fig. 2:**
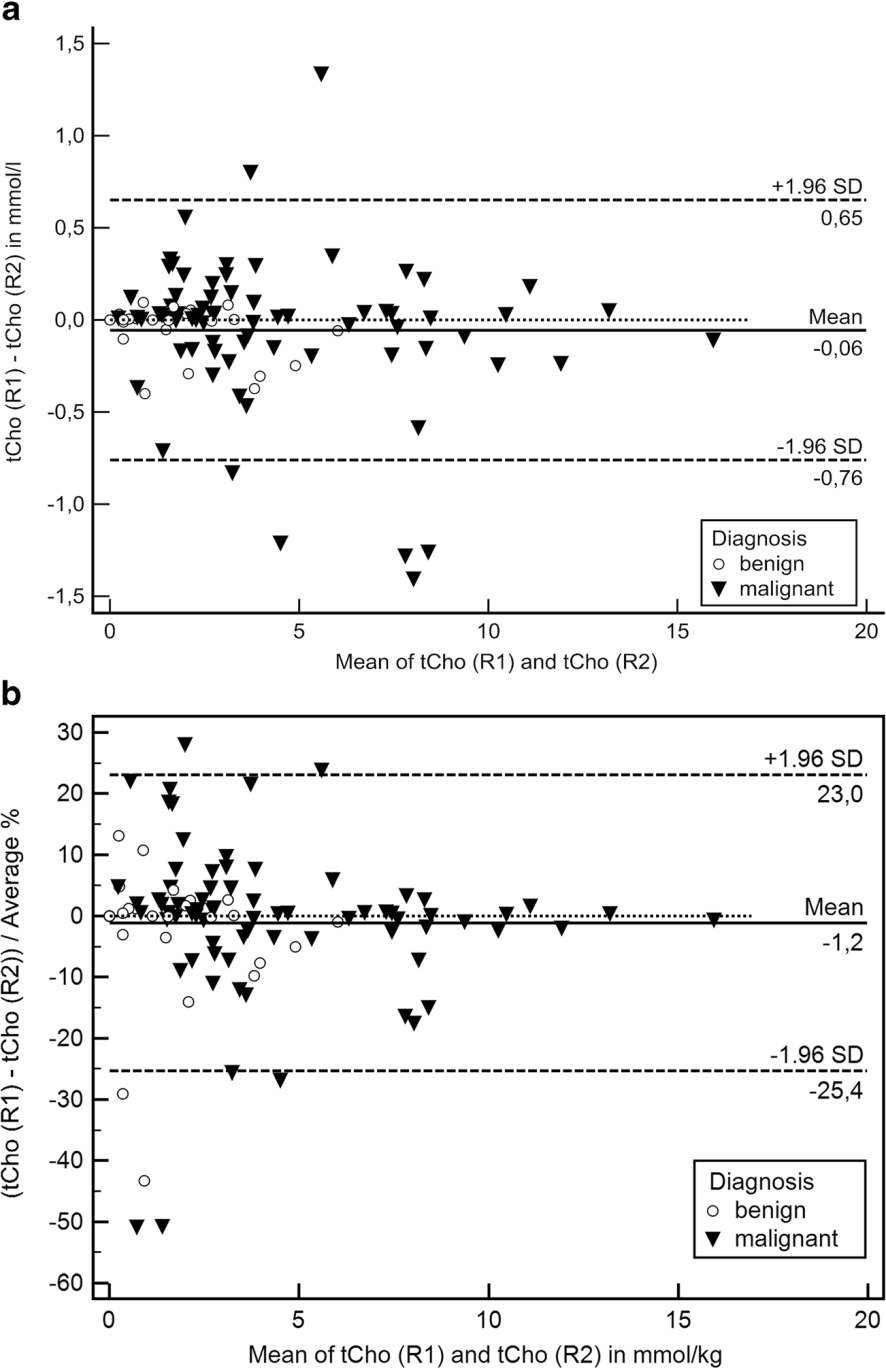
Bland-Altman plots of tCho differences between R1 and R2. **a** Absolute differences; **b** relative differences

Figure 2 demonstrates Bland-Altman plots for differences and limits of agreement between R1 and R2 as absolute (Fig. 2a) and percentage (Fig. 2b). The absolute mean difference between R1 and R2 was − 0.06 mmol/l, the limits of agreement ranging between − 0.76 and 0.65 mmol/l. The relative mean difference between R1 and R2 was − 1.2%, the limits of agreement ranging between − 25.4 and 23.0%.

### tCho to distinguish benign from malignant breast lesions

**Fig. 3:**
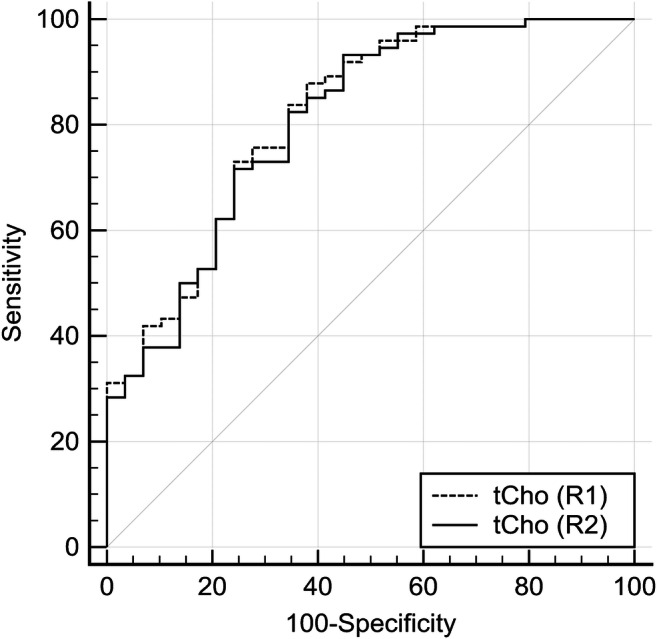
Receiver operating characteristic (ROC) curves estimating diagnostic performance of tCho to distinguish benign from malignant breast lesions (*n* = 103). Lesion volume serves as a reference and did not predict the presence of cancer (*p* > 0.05)

Figure 3 and Table 2 display the results of the ROC analysis. tCho showed a good area under the curve to distinguish benign from malignant lesions as measured by the AUC (0.816 (R1) and 0.809 (R2), *p* < 0.0001 for R1 and R2, respectively). The ROC curve analysis revealed that if tCho was ≤ 0.8 mmol/l, sensitivity for detection of breast cancer exceeded 95% for both readers (also see supplementary figure 2). Applying this threshold, could have potentially avoided *n* = 14/29 (48.3%, R1) or *n* = 13/29 (44.8%, R2) unnecessary biopsies in the investigated cohort while yielding *n* = 3/74 (4.1%, R1) or *n* = 2/74 (2.7%, R2) false negative diagnoses (two T1b hormonal receptor positive NST G2, one T1c hormonal receptor positive invasive tubular carcinoma G1).

**Table 2:** Diagnostic performance estimates and cutoff values for tCho to distinguish benign (*n* = 29) from malignant (*n* = 74) breast lesions

Parameter	AUC	*p* value	tCho cutoff in mmol/l	Sensitivity (95% CI) in %	Specificity (95% CI) in %
tCho (R1)	0.816	< 0.0001	0.8	96.0 (88.6–99.2)	48.3 (29.4–67.5)
tCho (R2)	0.809	< 0.0001	0.8	97.3 (90.6–99.7)	44.8 (26.4–64.3)

### tCho to predict positive lymph node metastasis

**Fig. 4:**
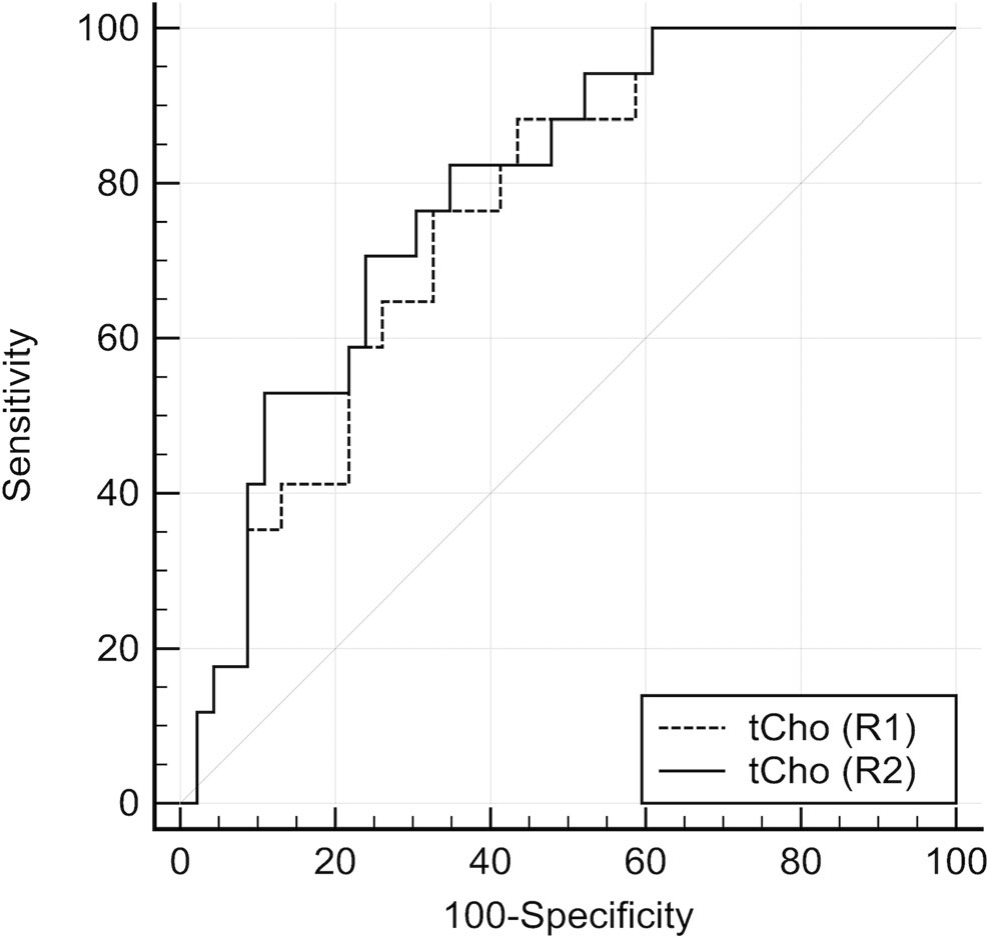
Receiver operating characteristics (ROC) curves estimating diagnostic performance of tCho to predict the presence or absence of metastatic lymph nodes (*n* = 63). Lesion volume serves as a reference and did not predict the presence of lymph node metastases (*p* > 0.05)

**Fig. 5:**
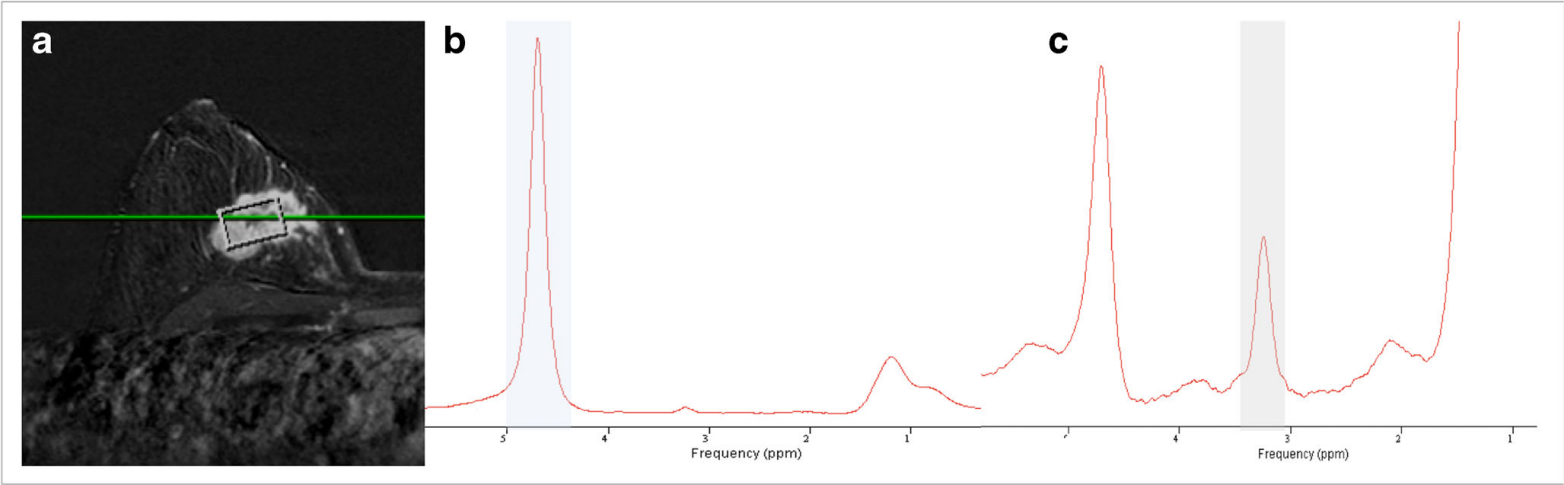
Thirty-four-year-old woman presenting with an enhancing suspicious mass in the early contrast enhanced subtraction image (**a**). **b** presents the water resonance peak at 4.74 ppm in the unsuppressed water reference spectrum (blue bar), weaker resonance peaks can be depicted at 3.23 (corresponding to total Choline tCho) and 1.3 and 0.9 ppm (methylene and methyl groups from lipids). The magnified water suppressed spectrum reveals the distinct tCho resonance peak at 3.23 ppm (gray bar). Using water as an internal reference, tCho was calculated as 13 mmol/l. Histopathology revealed an invasive ductal cancer NST G3 with ipsilateral lymph node metastases

**Fig. 6:**
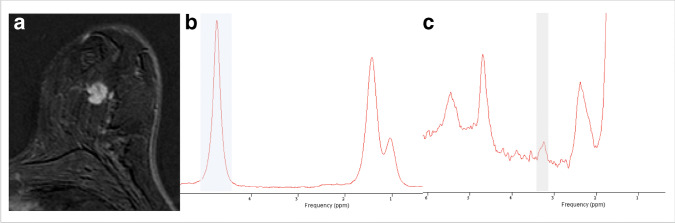
Forty-six-year-old woman presenting with an enhancing suspicious mass in the early contrast enhanced subtraction image (**a**). **b** presents the water resonance peak at 4.74 ppm in the unsuppressed water reference spectrum (blue bar). The magnified water suppressed spectrum reveals a weak tCho resonance peak at 3.23 ppm (gray bar). Using water as an internal reference, tCho was calculated as 1.9 mmol/l. Histopathology revealed an invasive ductal cancer NST G2 and negative lymph nodes

Figure 4 and Table 3 summarize ROC analysis results. Primary lesion tCho could significantly predict lymph node status with an AUC of 0.760 (R1, *p* < 0.0001) and 0.788 (R2, *p* < 0.0001). Below a tCho of 2.4 mmol/l, no metastatic lymph nodes where observed, thus achieving a sensitivity of 100% in both readers (also see supplementary figure 3). This condition applied to 39.1% (18/46) of all non-metastatic cancers in both R1 and R2. Details are given in Table 3. Clinical example cases are shown in Fig. 5 and Fig. 6.

**Table 3:** Diagnostic performance estimates and cutoff values for tCho to predict the presence (*n* = 17) or absence (*n* = 43) of metastatic lymph nodes

Parameter	AUC	*p* value	tCho cutoff mmol/l	Sensitivity (95% CI) in %	Specificity (95% CI) in %
tCho (R1)	0.760	< 0.0001	2.4	100 (80.5–100)	39.1 (25.1–54.6)
tCho (R2)	0.788	< 0.0001	2.4	100 (80.5–100)	39.1 (25.1–54.6)

## Discussion

Quantitative tCho evaluation from ^1^H-MRS allowed to diagnose malignancy and lymph node status in breast lesions that were considered suspicious with multiparametric breast MRI assessment using DCE, T2w and diffusion-weighted (DW) images. Specifically, low tCho concentrations associated with a low risk of breast cancer and lymph node metastasis. If confirmed by prospective studies, these findings would be of high clinical relevance as positive findings on MRI require invasive workup in order to avoid unnecessary surgery [24]. Further, the availability of MRI-guided interventions is limited [25], stressing the need for non-invasive tools to avoid unnecessary biopsies. While lymph node status is one of the most important predictors of breast cancer outcome, surgical management is increasingly conservative, avoiding axillary lymph node dissection in case of limited disease [26–28]. Again, a non-invasive tool to accurately assess the risk of present lymph node metastases could facilitate the clinical workup of breast cancer patients.

We were not the first group to investigate quantitative tCho measurements using an internal reference to distinguish benign from malignant breast lesions (see Table 4). Prior reports differed regarding their results, either reporting very high sensitivity and specificity [29, 30], very high specificity but moderate sensitivity [31], and only one group reported moderate sensitivity and specificity [32]. These different results can both be explained by the application of different thresholds and different patient and lesion groups. All groups examined lesions above 0.7 cm in size [29–32] and two reports pre-defined a minimum size of the investigated lesions and focused on mass lesions only [29, 31]. Our specificity is seemingly lower than previously reported (see Table 4). This is due to the different study design: opposed to prior reports, our study investigated whether MR spectroscopy can further improve diagnosis of suspicious mpMRI findings. The specificity values at the tCho threshold associated with a high sensitivity can thus be directly translated into the rate of avoidable biopsies of benign lesions [23]. Our results are possibly best compared to those of Dorrius et al, who investigated a comparable setting. In their study, the authors used ^1^H-MRS as an additional test to rule out cancer in a problem-solving setting using a three-dimensional spatially resolved spectroscopy technique [33]. We did not in detail investigate the effects of single techniques on diagnostic outcomes as reported by Pinker et al [34]. This was due to the fact that the study rationale was using MR spectroscopy only in case of suspicious multiparametric MRI using T2w, DWI, and DCE sequences. While several formal approaches of integrating these data into a diagnosis have been reported [35–38], our study protocol relied on the classical empirical approach of assigning BI-RADS categories. Our study design, however, allows to estimate the potential downgrade rates of benign breast lesions appearing suspicious upon triparametric (T2w, DWI, DCE) MRI as > 40%.

**Table 4:** Summary of prior reports on tCho quantification based on single-voxel acquisitions and comparison with the results reported in this paper

First author/year	Field strength	Cases	Cancer prevalence (%)	Sensitivity (%)	Specificity (%)	tCho* range
Thakur/2011 [1]	1.5 T	88	64.8	96.5	93.5	0–47.1
Baek/2012 [2]	1.5 T	112	88.4	65.7	92.3	0.9–10
Sah/2012 [3]	1.5 T	189	79.9	76.2	76.3	^+^0.58/1.6/4.2–5.4
Suppiah/2012 [4]	1.5 T	57	73.7	95.2	93.3	0.1–6.9
This study/2018	1.5 T	103	71.8	95.9	48.3^#^	0–16

* in mmol/l; ^+^[1–4] no range given. Values indicate mean values for healthy tissue, benign and malignant lesions. Malignant lesions were reported in subgroups. ^#^This study investigated only lesions classified as BI-RADS 4 or BI-RADS 5 after full triparametric (T2w, DWI, DCE) breast MRI assessment. Thus, the reported specificity does only apply to tCho quantification as investigated in this specific situation

We observed higher tCho levels in hormonal receptor–positive breast cancers. This seems to be in line with reports on choline kinase deregulation promoting estrogen receptor-driven proliferation [39]. Similar findings have been reported by Sah et al [32]. While the significance of this finding, e.g., on the general applicability of the exploratory tCho thresholds provided within this study cannot be estimated due to the limited sample size of our study (all FN findings were hormonal receptor positive), implications arise regarding the potential prediction of antihormonal treatment response. The tCho concentrations in our study fall within the range reported in prior studies (see Table 4). Note, however, that though using water as an internal reference addresses several normalization aspects such as voxel size and coil sensitivity differences, the method applied here and in the referenced papers relies on fixed assumptions regarding the real water concentration in tissue as well as the relaxation times of water and tCho in vivo [20]. Individual variations of these parameters, in particular tissue water concentration that might vary due to patient hydration, age, treatment and hormonal status, likely explain differences regarding tCho concentrations reported in the individual studies. We demonstrated a good reproducibility of tCho quantitation using the AMARES method. Though important in the field of imaging biomarker research, this aspect has only been investigated in other techniques such as breast DWI [40]. Due to the clinical study design, we did not analyze the repeatability of ^1^H-MRS data acquisition and inter-scanner variability. In this context, individual measurements of tCho and water relaxation times might be desirable in order to assess their potential impact and weigh it against the additional measurement time required. Therefore, all quantitative thresholds must be regarded as exploratory only until independently validated. Another issue is the potential interaction of tCho with contrast media that is further outlined below.

Our study results further indicate that the primary breast cancer tCho levels are predictive for lymph node metastases. Specifically, a low tCho concentration indicated the absence of lymph node metastases in a substantial proportion of our breast cancer cases. If prospectively validated, sentinel node biopsies (SNB) could be potentially avoided in patients classified as being at low risk for lymph node metastasis. While anecdotal and encouraging evidence exists on direct ^1^H-MRS in axillary lymph nodes [41], a direct measurement would require visibly enlarged lymph nodes and a time-consuming additive measurement. Indirect axillography based on an imaging phenotype of the index breast cancer has been shown by Dietzel et al in a radiomic approach using artificial neural networks on semantic lesion features [42]. Whether ^1^H-MRS data can contribute to prognostic breast cancer models or adds to morphological lymph node assessment remains to be investigated.

Our analysis is limited regarding several aspects: first, our spectroscopy technique is not spatially resolved. Though prior reports have reported encouraging results on spatially resolved ^1^H-MRS, quantification is still not established and difficult to achieve within a non-research setting. In addition, while theoretically of benefit, a spatially resolved spectroscopy still can only cover a region of interest and not two breasts. In the investigated application of ^1^H-MRS, that is the downgrading of lesions suspicious on conventional MRI assessment, it is of limited relevance to yield spatially resolved images as the area of interest is directly investigated. In this respect, our study is unique as we decided to use ^1^H-MRS on the spot, that is while the patient was still in the scanner. We are well aware that in many institutions MRI scans are acquired and interpreted at different time points and even different physical locations. While our approach may thus be difficult to employ in some institutions, the results do still apply: ^1^H-MRS has an additional diagnostic value in mpMRI of the breast and provides additional, prognostically relevant information on lymph node status. The PRESS sequence used had a long echo time. While shorter echo times may increase the tCho signal, TE is considered in the quantitation procedure. Besides, a meta-analysis reported no influence of TE on diagnostic performance of Cho assessment by ^1^H-MRS [16]. One potential drawback is the need for IV contrast. Gd-based contrast media show an interaction with the tCho signal in vivo [43], an effect that has been reported as being more pronounced in ionic contrast media and thus must be considered when comparing different study results [44]. Acquisition time is a further limitation of the used approach. Though aiming at screening, there is a trend to reduce magnet times in breast MRI and an additional test must prove its value also regarding magnet time efficiency [45]. Modern 3-T systems in combination with multichannel coils could substantially reduce acquisition times due to SNR gain that is more than fourfold [46] while a reduction of averages by 50% would only reduce the SNR by square root of 2. Consequently, the acquisition times reported here could potentially be reduced to 3 min or less while yielding the same SNR.

In conclusion, quantitative tCho evaluation from ^1^H-MRS allowed to diagnose malignancy and lymph node status in breast lesions that are considered suspicious with multiparametric breast MRI assessment using DCE, T2w, and diffusion-weighted (DW) images. tCho demonstrated the potential to improve diagnosis of malignancy in breast MRI lesions suspicious on mpMRI and stratify the risk of lymph node metastases with the aim of improved patient management.

## Electronic supplementary material

ESM 1 (DOCX 317 kb)

## Abbreviations

^1^H-MRS, MRS: Proton MR spectroscopy
ADC: Apparent diffusion coefficient
AUC: Area under the curve
BI-RADS: Breast imaging–reporting and data system
DCE: Dynamic contrast enhanced
DWI: Diffusion-weighted imaging
FLASH: Fast low angle shot
GRAPPA: GeneRalized Autocalibrating Partial Parallel Acquisition
IRB: Institutional Review Board
MRI, mpMRI: Magnetic resonance imaging, multiparametric MRI
PRESS: Point Resolved Spectroscopy Sequence
ROC: Receiver operating characteristics
tCho: Total choline
TE: Echo time
TR: Repetition time
TSE: Turbo spin echo
