# Supplementary figures and images for: Clinical relevance of total choline (tCho) quantification in suspicious lesions on multiparametric breast MRI
Source: Eur Radiol. 2020 Feb 17;30(6):3371–82. doi: 10.1007/s00330-020-06678-z (PMC7248046; doi:10.1007/s00330-020-06678-z)

**Supplementary Materials**

**Supplementary Figure 1**


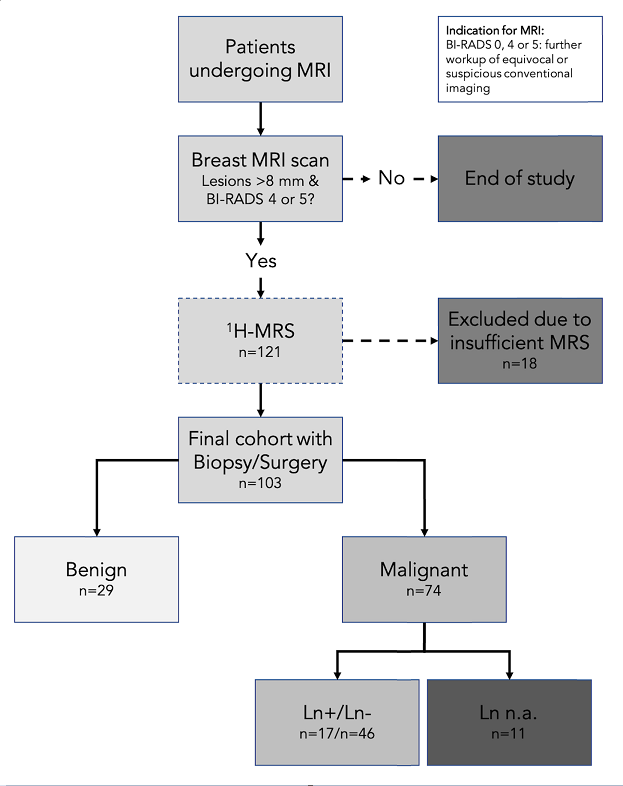


**Supplementary Figure 2**


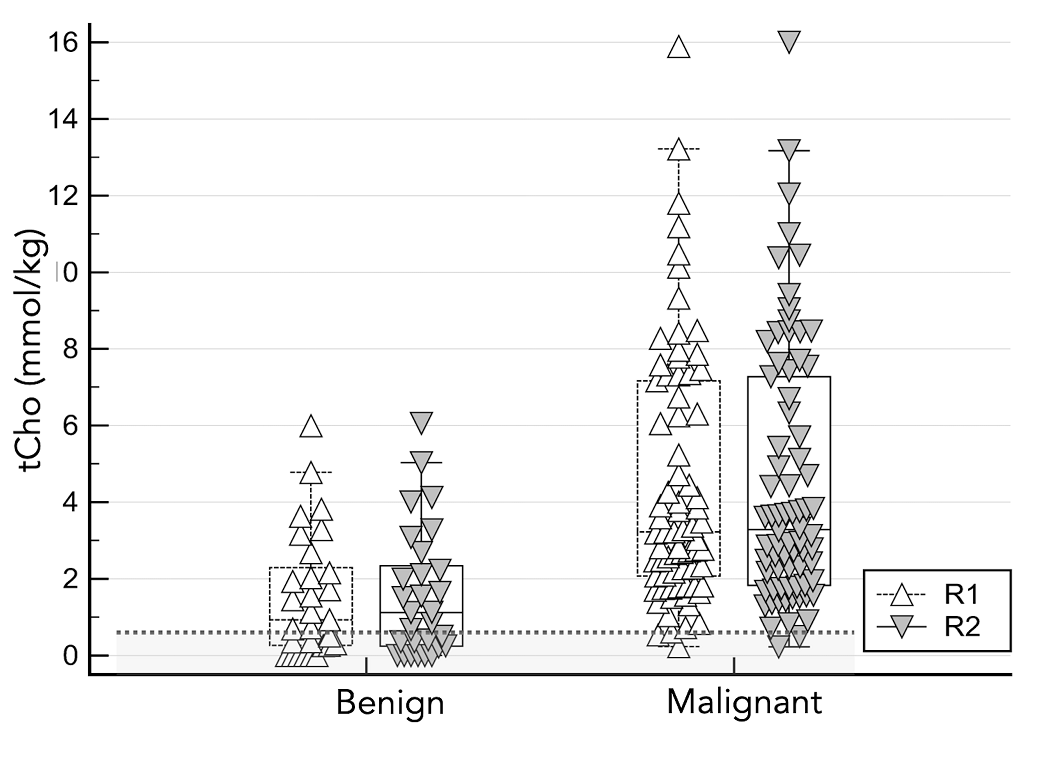


**Supplementary Figure 3**


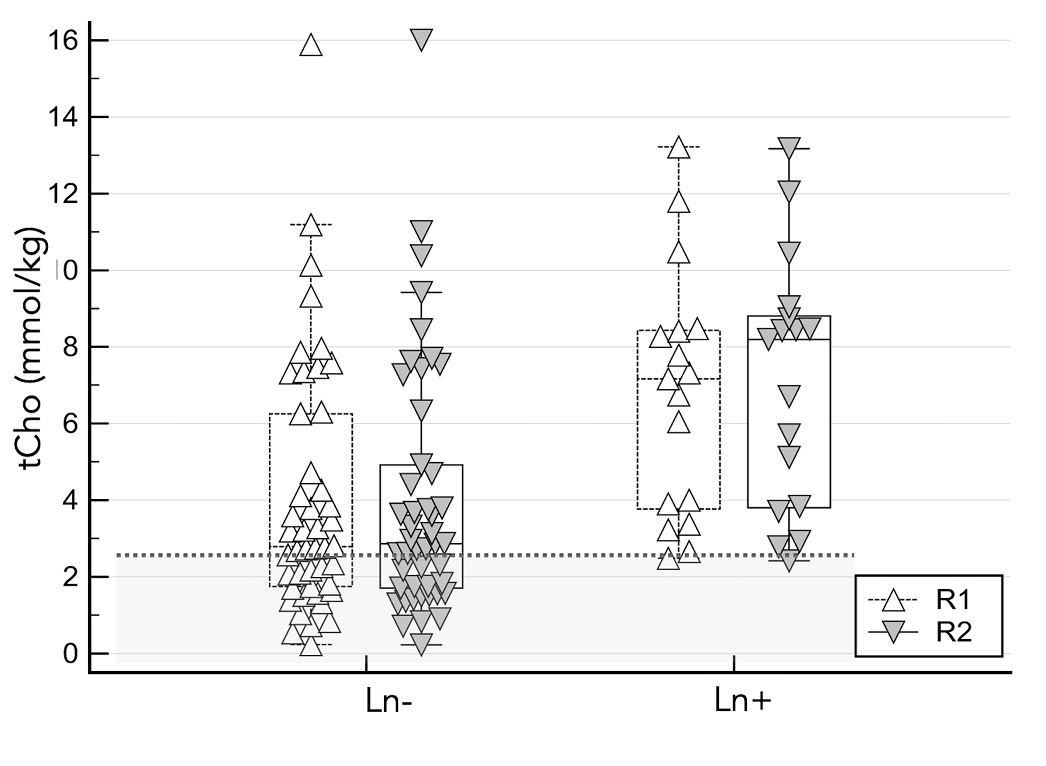

Supplement: Supplementary file 1 — (DOCX 317 kb) [file 330_2020_6678_MOESM1_ESM.docx]
